# Normal Variants, Pitfalls and Artifacts in Ga-68 DOTATATE PET/CT Imaging

**DOI:** 10.3389/fnume.2022.825486

**Published:** 2022-02-07

**Authors:** Nico Malan, Mboyo-Di-Tamba Vangu

**Affiliations:** Division of Nuclear Medicine and Molecular Imaging, Department of Radiation Sciences, University of the Witwatersrand, Johannesburg, South Africa

**Keywords:** neuro-endocrine tumors, positron emission tomography/computer tomography, Ga68-DOTATATE, variants, pitfalls, normal biodistribution, PET/CT

## Abstract

Indium 111 DTPA Octreotide (Octreoscan) has been the pillar of Somatostatin receptor (SSTRs) imaging in nuclear medicine for over three decades. The advent of PET/CT brought new analogs of somatostatin that have higher affinity and improved resolution due to their labeling to Gallium 68 for positron imaging. The most used analogs include DOTATATE, DOTATOC and DOTANOC. However, Gallium 68–1,4,7,10-tetraazacyclododecane-1,4,7,10-tetraacetic acid (DOTA)-octreotate (DOTATATE) is probably the most common non-FDG (fluoro-2-deoxy glucose) PET tracer alongside PSMA (prostate specific membrane antigen). In contrast to F18-labeled FDG, it does not require proximity to a cyclotron due to the availability of the Ga68 generator. DOTATATE is a somatostatin analog which allows whole body imaging of somatostatin receptors on cell surfaces. 68Ga-DOTA compounds provide the imaging standard for well-differentiated (Grade 1 and low grade 2) neuro-endocrine tumors (NETs) and is utilized in the staging and characterization and restaging of patients with NETs. 68Ga DOTATATE has a complementary role with 18F-FDG where tumors may exhibit varying degrees of differentiation. It furthermore has application as a prelude to therapy in selecting patients for peptide receptor radionuclide therapy using a theranostic approach. A sound knowledge of the normal biodistribution of the radiotracer is imperative for optimal patient outcome and to avoid potential false positives such as inflammation, normal pancreatic uncinate process uptake and osteoblastic activity. In this review, we will describe the normal appearances of the 68Ga DOTATATE and the potential pitfalls with the support of images to aid in improving interpretation of this crucial innovative tool in the management of individuals with tumors expressing SSTRs.

## Introduction

Neuroendocrine tumors form part of a heterogeneous group of tumors that originate from neuroendocrine cells. They are found in various organs and frequently involve cells of the gastrointestinal tract and the lungs. Organs that are less frequently involved include organs with endocrine function: thyroid gland, parathyroid, adrenal medulla as well as the pituitary gland. Neuroendocrine cells typically express somatostatin receptors (SSTR) on the cell surfaces and it is this attribute that provides a specific target for molecular imaging.

Single photon tracers have been the mainstay of neuroendocrine tumor imaging ever since the advent of synthetically produced Octreotide. The significantly longer half-life of Octreotide (90 min vs. a few minutes for naturally occurring somatostatin) enabled imaging when labeled to single photon emitters such as Indium-111. Through planar whole-body images as well as dedicated SPECT/CT reconstructed images of the abdomen and / or thorax diagnostic images were acquired.

The advent of positron emission tomography/computer tomography (PET/CT) and specifically the 68Ge/68Ga generator revolutionized imaging of neuroendocrine tumors. PET/CT imaging where available has replaced imaging with 111In-octreotide due to its higher specificity as well as the improved resolution owing to positron emission tomography.

68Ga is a generator produced short lived (half-life of 68 min) isotope and is typically chelated to 1,4,7,10-tetraazacyclododecane-1,4,7,10-tetraacetic acid (DOTA) which typically shows higher affinity than the chelator for single photon emitters. This in turn is then bound to a peptide. There are various peptides available (DOTATOC, DOTANOC, DOTATATE). These peptides exhibit affinity to various somatostatin receptor subtypes. There are five known subtypes of Somatostatin receptors (SSTR) on cell surfaces. SSTR 1 to SSTR 5. DOTATATE has the highest affinity to SSTR2 and SSTR2 which are commonly expressed in neuroendocrine tumors (1–6).

## Normal Biodistribution

Physiological distribution of 68Ga-DOTATATE is attributed to both the presence of somatostatin receptors (specifically SSRT2) as well as non-specific clearance of the tracer.

The most avid uptake is noted in the spleen, adrenal glands, kidneys, and pituitary gland.

The liver, thyroid gland and salivary glands demonstrate moderate uptake.

**Figure 1:**
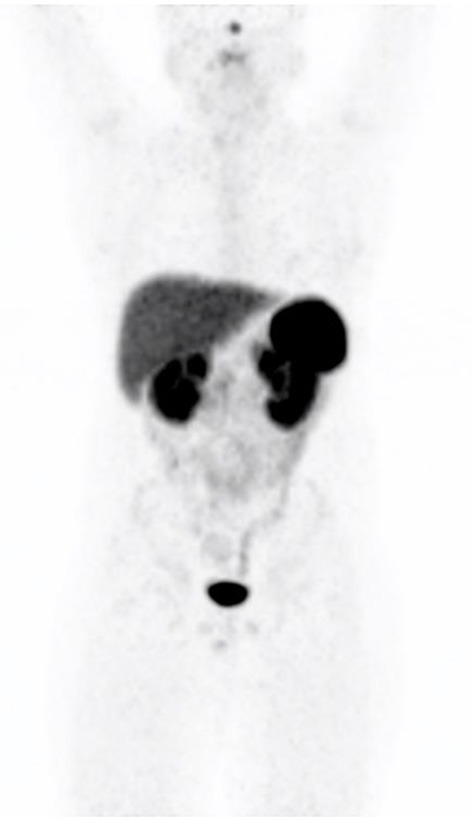
Maximal intensity projection image demonstrating the normal biodistribution of 68Ga-DOTATATE.

The tracer, being hydrophilic undergoes renal excretion where the small peptide molecule undergoes filtration and partial reabsorption in the proximal tubule (see Figure 1).

The liver, and salivary gland activity is due to non-specific tracer handling.

Lungs have very little or any activity as there are mainly SSTR4 receptors present in the lungs. Splenic uptake is due to the SSTR2 receptors that are found mainly in the red pulp of the spleen. The uncinate process of the pancreas has a high density of SSTR2, 3 and 5 receptors which explains the finding of increased activity in the uncinate process. Breast tissue and the thyroid gland may accumulate low and diffuse activity which is related to the presence of SSTR2 receptors. Gastro-intestinal, especially the fundus of the stomach and mucosa of the intestines may show activity and is also related to the presence of SSTR2 receptors.

**Figure 2:**
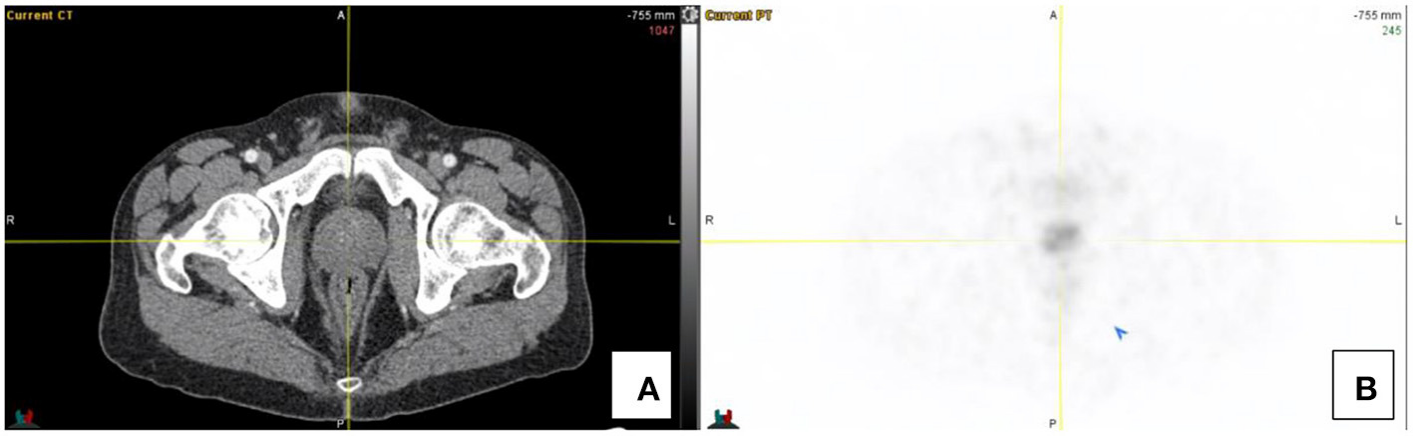
Transaxial CT **(A)** and PET images **(B)** localizing low grade diffuse activity in the prostate gland (SUV_max_ 2.8).

Low grade activity (7) may be appreciated in the prostate gland and should be uniform, *in vitro* studies have shown SSTR2 receptors in the smooth muscle of the stroma of the prostate gland but not in the glandular tissue (see Figure 2). SSTR2 receptors are also found on macrophages and bone marrow hematopoietic precursors which explains some low grade vascular and bone marrow activity.

68Ga-DOTATATE images typically show very low background activity in the soft tissues thus yielding high target to background images. A so-called “sink effect” has been described whereby there may be decreased physiological uptake reflecting high burden of disease with increased uptake in pathological sites (1–6, 8, 9).

## 68GA-Dotatate and Tumor Grade

The World Health Organization graded neuroendocrine tumors according to immunohistochemical staining of a marker of cellular proliferation, Ki-67. Lower grade tumors show differentiation and high-grade tumors show dedifferentiation.

68Ga-DOTATATE and 18F fluoro-2-deoxy-d-glucose (FDG) have a complementary role in imaging neuroendocrine tumors. A so-called “flip-flop” effect is attributed to tumor heterogeneity whereby there may be a spectrum of disease, well-differentiated lesions (G1) may co-existing with poorly differentiated lesions (G3). G2 tumors may demonstrate either DOTATATE or FDG uptake (1–6, 8, 9).

It is important to keep in mind that neuroendocrine neoplasms may involve quite a number of sites and thus it is important to recognize this as pathology and not false positives. Table 1 provides a summary of the site specific classification of the International Agency for Research on Cancer (IARC) and World Health Organization (WHO) expert consensus proposal. Note that neuroendocrine neoplasms may form part of syndromes, amongst others, multiple endocrine neoplasia type 1 (MEN1—pituitary adenoma, parathyroid hyperplasia and pancreatic tumors) and multiple endocrine neoplasia type 2 (MEN2—parathyroid hyperplasia, medullary thyroid carcinoma and pheochromocytoma) (10).

**Table 1:** Summary of a site specific classification of neuroendrocrine neoplasms.

Pancreatic and gastro-intestinal tract
Lung
Pituitary
Head and neck, including thyroid (medullary thyroid carcinoma) and parathyroid
Breast
Genito-urinary system and male and female genital organs
Adrenal and paraganglia
Skin


### Artifacts

PET/CT images are prone to artifacts. Artifacts may be encountered when there is misregistration between the anatomical CT images and the functional PET images. This may occur because of patient or respiratory motion.

Patient motion generally affects the extremities—head and neck, arms including the axillae and legs. Respiratory motion has its greatest effect on structures near the diaphragm.

**Figure 3:**
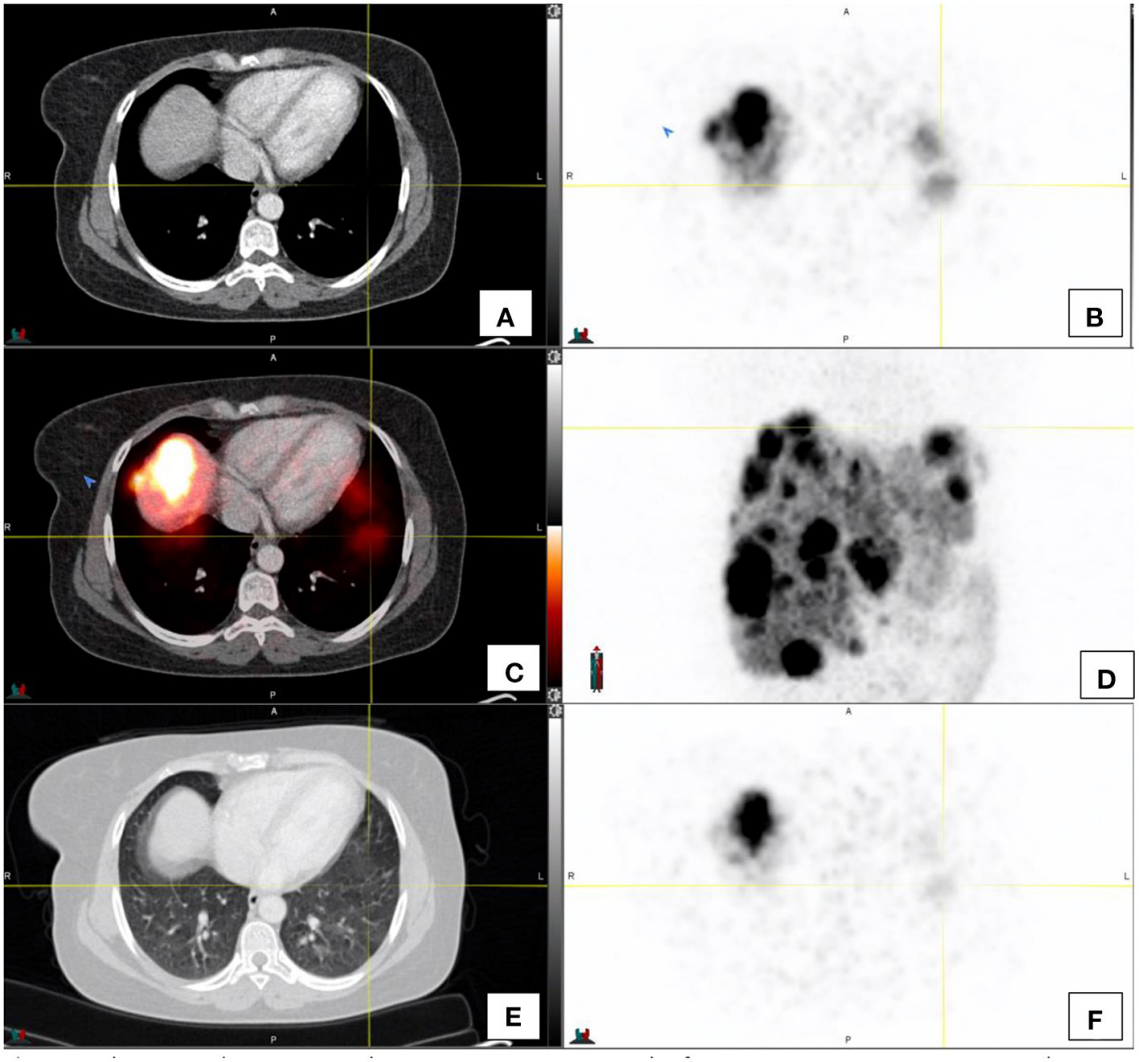
This image demonstrates the misregistration as a result of respiratory motion in a patient with hepatomegaly and extensive liver metastases in both the left and right lobes of the liver. Activity appears to be in relation to the lung [**(A–C)**—transaxial CT, PET and fused PET/CT images respectively], however the activity on PET **(F)** on CT window of the same region **(E)** does not show focal lung lesions. **(D)** shows the maximal intensity projection image of the region.

PET images are longer acquisitions where breath hold is not possible. The resultant PET images are the average of tidal breathing. CT images are fast breath hold acquisition images. This may cause misregistration of the PET and CT images. The maximal intensity images (MIP images) should always be inspected to alert the reader to potential sites of increased avidity, and this should then be contextualized with the CT anatomical images (see Figure 3). It is worthwhile noting that respiratory motion artifacts which cause misregistration between PET and CT images can be minimized by using respiratory gated acquisitions.

With the CT component being used for attenuation correction, overestimation of activity in dense structures (such as metallic implants) should be guarded against (9).

### Potential Pitfalls

Many potential pitfalls relate to false positive findings. False negative findings usually relate to tumor heterogeneity, which was discussed earlier, where FDG PET/CT scans fulfill a complementary role in tumor heterogeneity where there may be lesions or part of the tumor that is dedifferentiated (9).

Hofman et al. (1) conveniently classified potential false positive pitfalls into the following categories:

1. Physiological
2. Osteoblastic
3. Inflammatory
4. Incidental

Physiological false positive pitfalls relate to the pancreas and spleen. The pancreas may demonstrate heterogeneous activity which is related to islet cell hypertrophy or prominent clusters thereof. This activity is usually low grade and should not be mistaken for disease.

**Figure 4:**
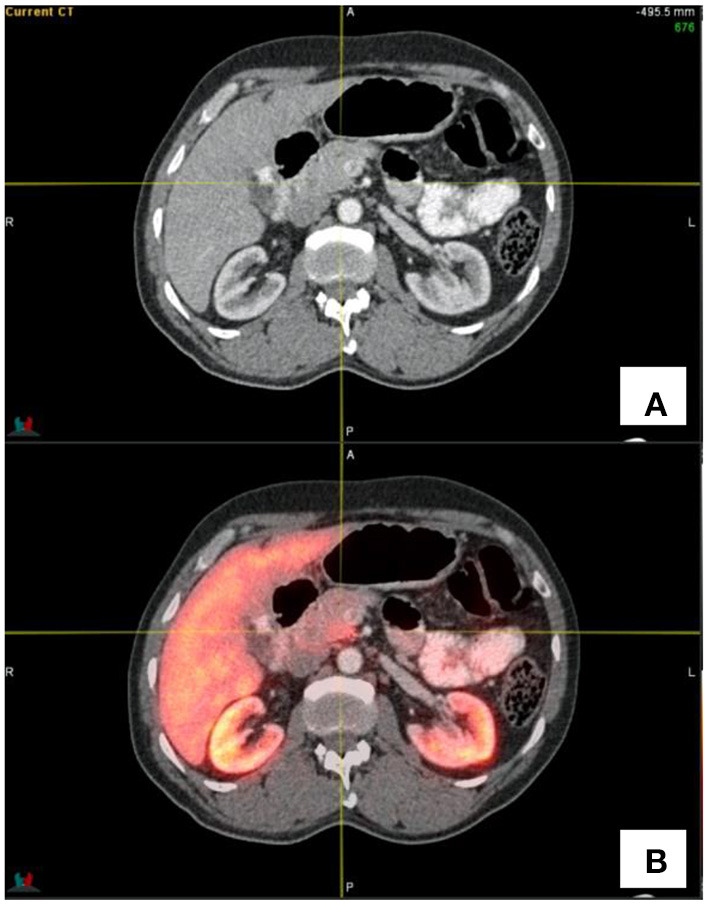
Transaxial CT **(A)** and fused PET/CT **(B)** images showing the increased activity in the uncinate process as indicated by the cross-hairs.

The uncinate process of the pancreas may often demonstrate increased activity in relation to the increased expression of SSTR2. This activity tends to be ill-defined and is seen in relation to the uncinate process of the pancreas (see Figure 4).

**Figure 5:**
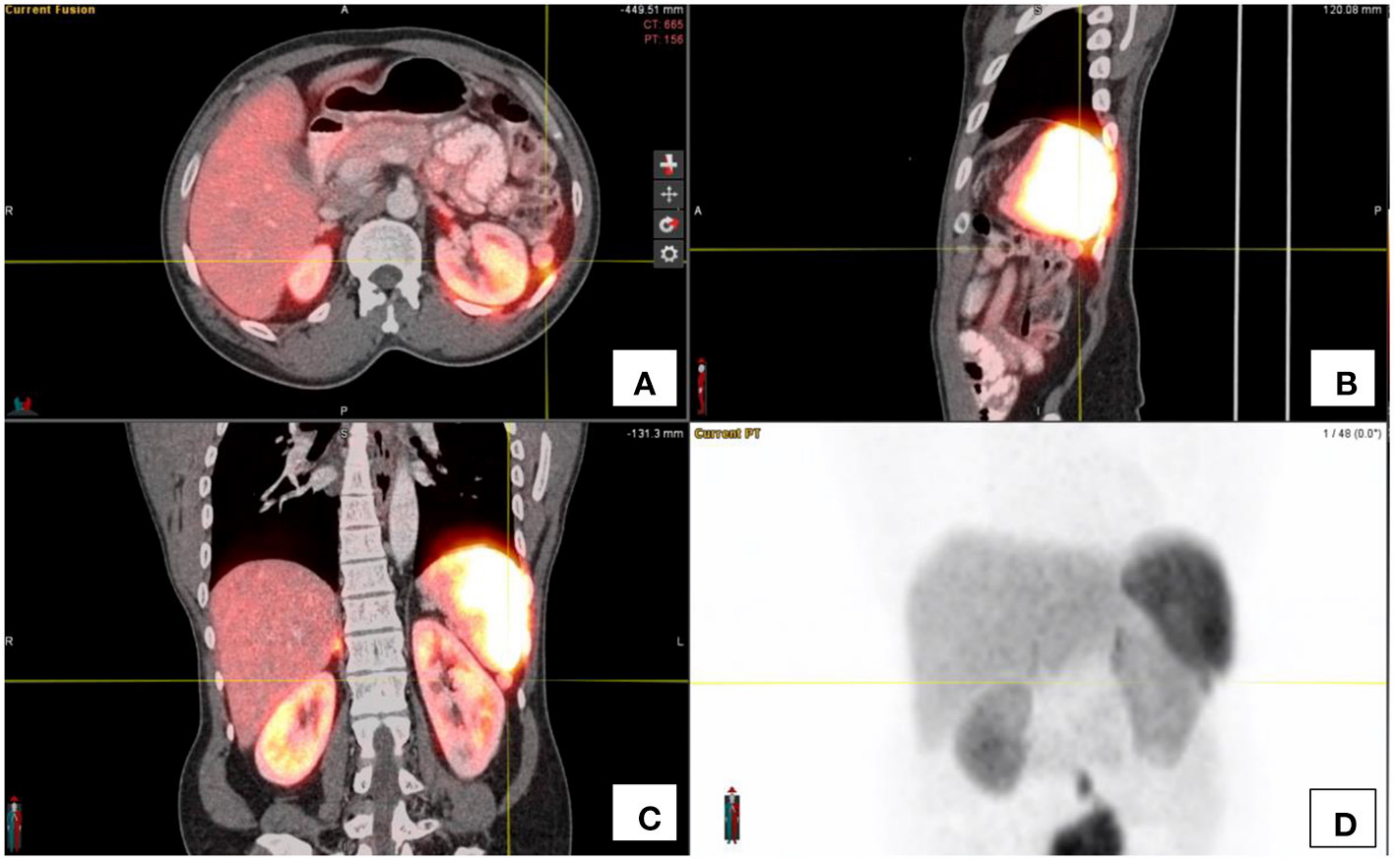
**(A–C)** show the fused PET/CT images of the spenuncle in the axial, saggital and coronal orientations respectively. The maximal intensity projection image **(D)** shows the focal activity below the spleen as indicated by the cross-hairs.

The spleen demonstrates intense Ga68-DOTATATE uptake. Thus, accessory spleens, splenunculi and splenosis following splenectomy may be sources of false positives (see Figure 5). A high clinical index of suspicion should assist, as well as comparing the enhancement on CT to that of the spleen. The addition of a denatured red blood cell scan may assist in doubtful instances, to classify lesions as splenic related lesions (1).

**Figure 6:**
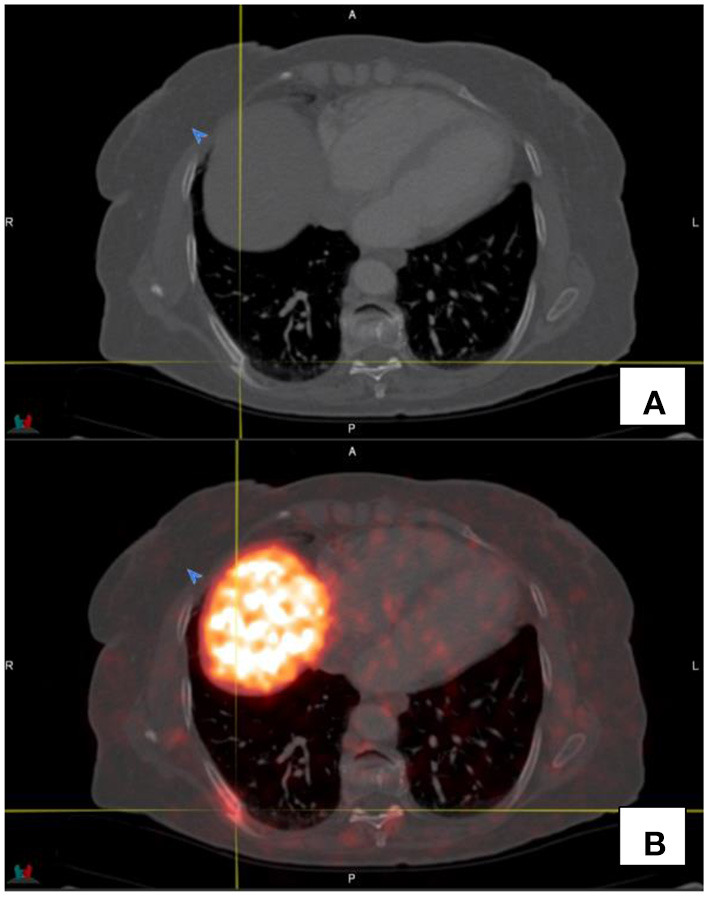
Transaxial CT **(A)** and fused PET/CT **(B)** images show a fracture of the right 7th rib with associated increased Ga68-DOTATATE uptake.

Active osteoblastic activity by virtue of osteoblasts expressing SSTR2 receptors, may also accumulate DOTATATE activity. These foci of uptake tend to be low-grade or moderate in intensity and the morphological findings on CT component will assist in the interpretation of these entities (1, 11). Osteoblastic processes that have been described to accumulate DOTATATE activity include: osteophytes, Schmorl's nodes, fractures, metabolic bone diseases such as Paget's as well as growth plates in younger patients (see Figure 6).

**Figure 7:**
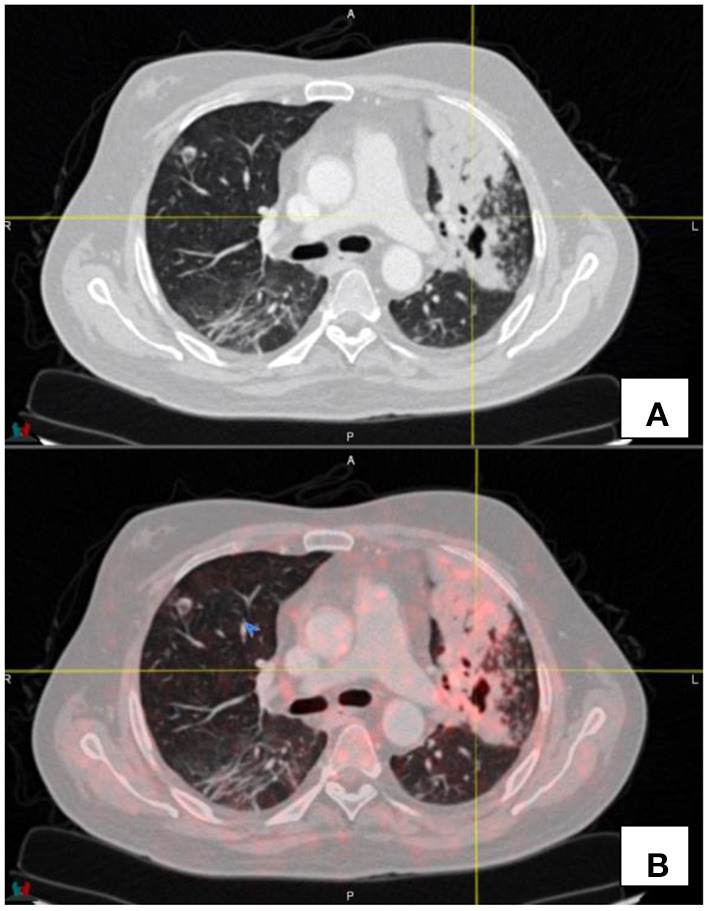
Transaxial CT **(A)** and fused PET/CT **(B)** images demonstrate low grade uptake (SUV_max_ 2.1) in a patient known with fibrocavitatory pulmonary tuberculosis—the patient was recently started on treatment for tuberculosis.

Active inflammatory processes have been shown to demonstrate DOTATATE uptake. This is not surprising since activated white blood cells and in particular macrophages and leukocyte do express SSTR2 receptors (1). This may result in low grade activity in reactive or inflammatory nodes, prostatitis, inflammatory change within the radiation field, sarcoidosis atherosclerotic plaques and in other inflammatory processes (see Figure 7).

There have also been case reports in the literature where 68Ga-DOTATATE activity was documented in benign gynecomastia. The uptake mechanism is thought to be non-specific (12).

A further case report showed 68Ga-DOTATATE activity in relation to pancreatic metastases of renal cell carcinoma which mimicked a pancreatic neuroendocrine tumor. The uptake is based on the expression of SSTR2 receptors in renal cell carcinoma (13).

Low grade activity has also been described in cervical stellate ganglions. This is typically seen at the cervicothoracic junction para-vertebral and should not be interpreted as pathological lymph nodes (14).

### Neuroendocrine Tumors and Treatment With Somatostatin Analogs

Haug et al. (15) showed that in a study of 105 patients where 35 patients were pre-treated with long-acting octreotide, the presence of long-acting octreotide did not significantly decrease 68Ga-DOTATATE tumor uptake but resulted in better tumor to background ratio through a reduction in liver, spleen, and thyroid activity. The same was shown by Ayati et al. (16) that assessed 30 patients that were scanned before and after commencing treatment with long-acting somatostatin analogs.

## Conclusion

68Ga-DOTATATE has become the new standard of imaging neuroendocrine tumors through a superior accuracy and high sensitivity as well as whole body assessment of somatostatin receptor expression.

Not only does 68Ga-DOTATATE and 18F-FDG fulfill a complementary role in instances where there is tumor heterogeneity, but Ga68-DOTATATE can also be used as a prelude to therapy when a theranostic approach is envisaged in patients where treatment options may be limited.

As shown, 68Ga-DOTATATE PET/CT imaging (see Table 2) is prone to interpretive pitfalls through technical factors as well as an array of potential false positive findings. A sound knowledge of these pitfalls and experience in reporting 68Ga-DOTATATE will improve diagnostic outcome as well as patient specific outcomes.

**Table 2:** Summary of potential artifacts and pitfalls that may be encountered with 68Ga-DOTATATE.

Artifacts:
Respiratory motion Patient motion
Pitfalls: False Negatives
Tumor dedifferentiation Tumor heterogeneity
Pitfalls: False Positives
Physiological: Pancreas islet cell hypertrophy/prominent clusters Uncinate process of the pancreas Splenuncle/Splenosis Gynaecomastia Cervical stellate ganglions
Osteoblastic: Osteophytes Schmorl's nodules Fractures Metabolic bone disease (for example Paget's) Growth plates in growing skeletons
Active inflammation: Reactive nodes Post radiation areas Atherosclerotic plaques Inflammatory diseases (sarcoidosis / tuberculosis) Prostatitis

## Author Contributions

All authors listed have made a substantial, direct, and intellectual contribution to the work and approved it for publication.

## Conflict of Interest

The authors declare that the research was conducted in the absence of any commercial or financial relationships that could be construed as a potential conflict of interest.

## Publisher's Note

All claims expressed in this article are solely those of the authors and do not necessarily represent those of their affiliated organizations, or those of the publisher, the editors and the reviewers. Any product that may be evaluated in this article, or claim that may be made by its manufacturer, is not guaranteed or endorsed by the publisher.
